# Bioclickable Mussel-Derived Peptides With Immunoregulation for Osseointegration of PEEK

**DOI:** 10.3389/fbioe.2021.780609

**Published:** 2021-11-24

**Authors:** Huan Zhao, Xiaokang Wang, Wen Zhang, Lin Wang, Can Zhu, Yingkang Huang, Rongrong Chen, Xu Chen, Miao Wang, Guoqing Pan, Qin Shi, Xichao Zhou

**Affiliations:** ^1^ Department of Orthopaedics, The First Affiliated Hospital of Soochow University, Orthopaedic Institute of Soochow University, Suzhou, China; ^2^ Department of Orthopaedics, The Affiliated Maternity and Child Health Care Hospital of Nantong University, Nantong University, Nantong, China; ^3^ Department of Pediatrics, The Affiliated Maternity and Child Health Care Hospital of Nantong University, Nantong University, Nantong, China; ^4^ Institute for Advanced Materials, School of Materials Science and Engineering, Jiangsu University, Zhenjiang, China

**Keywords:** PEEK, clickable mussel-biomimetic peptide, bone morphogenetic protein 2 functional peptides, bio-orthogonal reaction, osseointegration, FOXP3+ regulatory T cells

## Abstract

Polyether ether ketone (PEEK)–based biomaterials have been widely used in the field of spine and joint surgery. However, lack of biological activity limits their further clinical application. In this study, we synthesized a bioclickable mussel-derived peptide Azide-DOPA_4_ as a PEEK surface coating modifier and further combined bone morphogenetic protein 2 functional peptides (BMP2p) with a dibenzylcyclooctyne (DBCO) motif through bio-orthogonal reactions to obtain DOPA_4_@BMP2p-PEEK. As expected, more BMP2p can be conjugated on PEEK after Azide-DOPA_4_ coating. The surface roughness and hydrophilicity of DOPA_4_@BMP2p-PEEK were obviously increased. Then, we optimized the osteogenic capacity of PEEK substrates. *In vitro*, compared with the BMP2p-coating PEEK material, DOPA_4_@BMP2p-PEEK showed significantly higher osteogenic induction capability of rat bone marrow mesenchymal stem cells. *In vivo*, we constructed a rat calvarial bone defect model and implanted PEEK materials with a differently modified surface. Micro-computed tomography scanning displayed that the DOPA_4_@BMP2p-PEEK implant group had significantly higher new bone volume and bone mineral density than the BMP2p-PEEK group. Histological staining of hard tissue further confirmed that the DOPA_4_@BMP2p-PEEK group revealed a better osseointegrative effect than the BMP2p-PEEK group. More importantly, we also found that DOPA_4_@BMP2p coating has a synergistic effect with induced Foxp3+ regulatory T (iTreg) cells to promote osteogenesis. In summary, with an easy-to-perform, two-step surface bioengineering approach, the DOPA_4_@BMP2p-PEEK material reported here displayed excellent biocompatibility and osteogenic functions. It will, moreover, offer insights to engineering surfaces of orthopedic implants.

## Introduction

With extended life expectancy worldwide, the number of people suffering from bone and joint diseases and injuries is increasing year by year. Most of the patients need to receive internal fixation implants to restore the structure and function of damaged bones or joints (Dhawan et al., 2012; Chamoli et al., 2014). Polyether ether ketone (PEEK) is a colorless organic thermoplastic polymer in the polyaryletherketone series. Because of its high mechanical strength, high temperature resistance, chemical resistance, abrasion resistance and excellent biocompatibility, PEEK has been widely used in medical treatments, such as spinal implants, joint reconstruction, and dental and craniomaxillofacial procedures (Ghosh and Abanteriba, 2016). In the last few years, PEEK implants have been widely accepted in the human body over other traditional plastics and metals. By 2017, PEEK cages accounted for 68% of inter-body devices, representing a market of over 1 billion US$ in the United States alone. However, the common problem of PEEK implants is lack of biological induction capacity, and the biological inertness of PEEK may hinder specific cell adhesion and tissue response (for example, adhesion, signal conduction, and stimulation) during bone regeneration at the bone-implant interface, which in turn affects the osseointegration of PEEK.

Osseointegration is the early stage of the implant to form a direct and stable bone-implant connection, and it plays a very vital role in the biological and clinical success of the implants (Asri et al., 2017). Therefore, more and more clinicians and researchers have modified PEEK and its composite materials through physical, chemical, and biological methods to improve its biological activity and osteogenic properties, including blending modification, direct surface modification, and surface coating modification (Kurtz and Devine, 2007). At present, the modification methods of physical technology require a variety of complex technical procedures, such as thermal spraying, pulsed laser, ion sputtering, sandblasting, electrochemical method, and ion implantation, which require a lot of optimization experiments and complex facilities. Some biologically active molecules, such as peptides, proteins, growth factors, and even inorganic ions (Ca^2+^), can be used to modify PEEK materials through physical adsorption or covalent access. However, the current physical adsorption method can cause serious molecular leakage, while the chemical method requires complex chemical means and non-biologically compatible chemical molecules to bridge the active molecules. Thus, surface coating modification of PEEK simply and conveniently is of great clinical significance in orthopedics and dentistry (Shah et al., 2019).

Marine mussel organisms have recently attraction attention because of their adsorption capacity. The adhesion plaques formed by them are composed of six kinds of foot proteins (*Mytilus edulis* foot proteins, Mfps) (Minati et al., 2017). Mfps are rich in a variety of catechol amino acids (3,4-dihydroxy-L-phenylalanine, DOPA). DOPA and its substrate can easily form covalent and non-covalent bonding, coupled with the interaction between various catechu groups, enabling marine mussel organisms to be able to adsorb to the surface of almost all solid objects under moist conditions, such as rocks, ship bottoms, and cement (Lee et al., 2006; Zhao and Waite, 2006). DOPA can provide strong adhesion on both inorganic and organic surfaces (Saiz-Poseu et al., 2013; Wei et al., 2014), which shows a huge prospect for surface modification of medical materials (Ejima et al., 2013; Yang et al., 2020a).

Bioclickable conjugation is a novel and simple attachment method, which is based on the reaction of a diarylcyclooctyne moiety (DBCO) with an Azide-labeled reaction partner. Unlike conventional click chemistry (copper-catalyzed alkyne-azide cycloaddition, CuAAC), its reaction is rapid at room temperature and does not require copper ions, which is toxic to most organisms and can cause protein denaturation. The DBCO-azide copper-free click method has been widely used in antibody-peptide preparation, labeling, and conjugation between biomolecules and cells and cell tracking (Gong et al., 2016; Yoon et al., 2016; Xiao et al., 2020).

In this study, we developed a biomimetic peptide Azide-DOPA_4_ which is derived from mussel foot protein. With a strong and widely applicable catechol group adhesion effect, DOPA_4_ can form an adhesion layer on the surface of PEEK, which can effectively improve the cell adhesion ability of PEEK. Due to the terminal azide groups on DOPA_4_, this would enable subsequent integration of the biofunctional module by a second-step conjugation of the DBCO-capping biomolecules through the DBCO-Azide bioclick reaction. Bone morphogenetic protein-2 (BMP-2) is a well-characterized growth factor in that it can induce osteoblast differentiation and bone formation, which are also widely used in biomaterial tissue engineering (Khan and Lane, 2004). Therefore, we further combined typical DBCO-bearing BMP-2 functional peptides (BMP2p) through the DBCO-Azide bioclick reaction to generate DOPA_4_@BMP2p PEEK and enhance the biological activity of PEEK. We anticipate that this improved surface strategy based on mussel adhesion and bio-orthogonal conjugation would provide a means for surface bioengineering of PEEK with optional functions.

## Materials and Methods

### Materials

**SCHEME 1:**
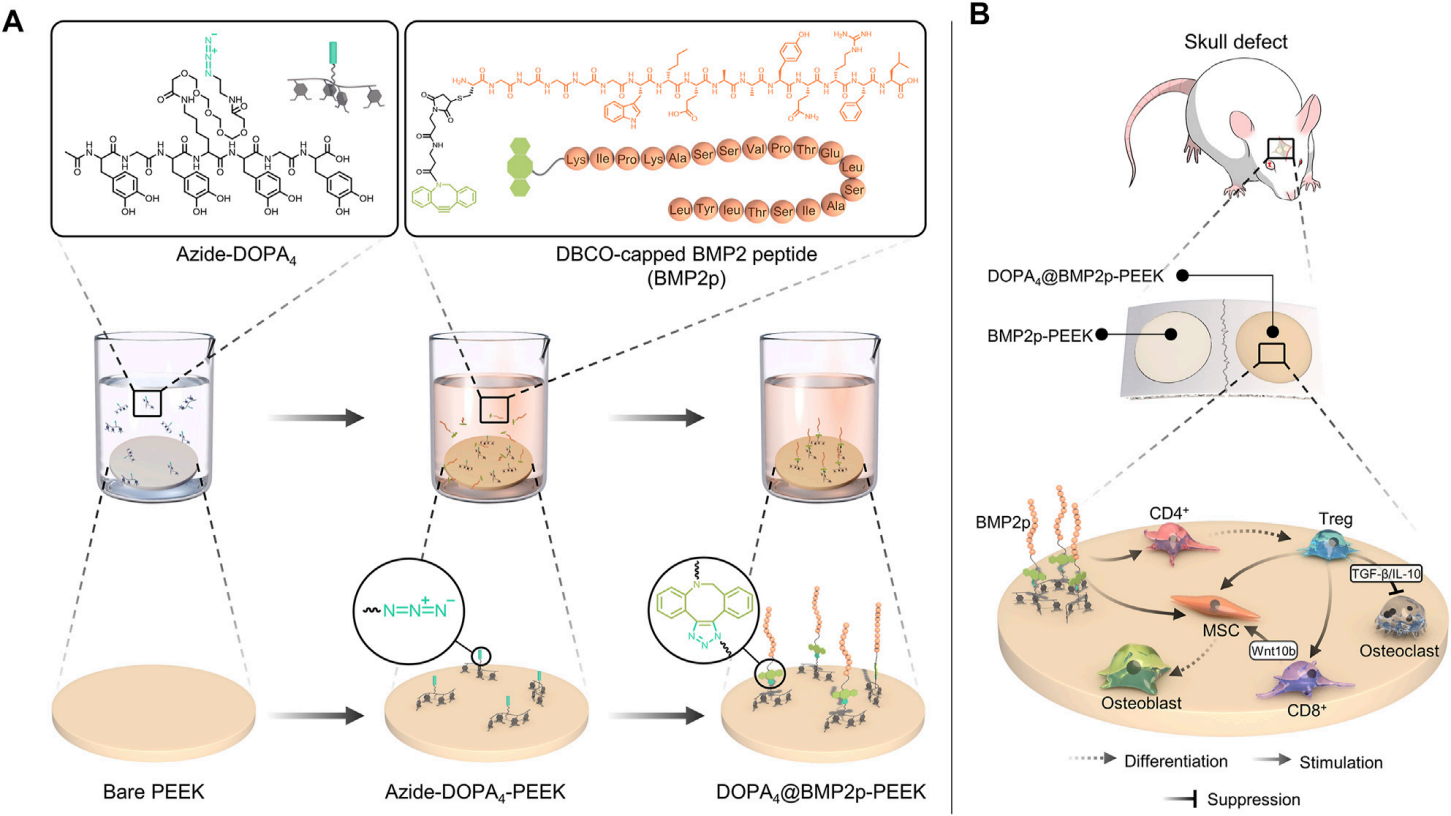
Schematic diagram of the two-step surface bioengineering of PEEK implants. **(A)** Upper: molecular formula of Azide-DOPA_4_ and bio-orthogonal conjugation of BMP2p and lower: schematic diagram of the surface modification of PEEK implants by bioclickable mussel-inspired Azide-DOPA_4_ and BMP2p. **(B)** Schematic diagram of osteogenic and immunoregulatory effects of the DOPA_4_@BMP2p PEEK material.

According to a previously reported method (Pan et al., 2016; Liu et al., 2018; Ma et al., 2019), Azide-DOPA_4_ (Scheme 1A), BMP2p (Lys-Ile-Pro-Lys-Ala-Ser-Ser-Val-Pro-Thr-Glu-Leu-Ser-Ala-Ile-Ser-Thr- Leu-Tyr-Leu, Scheme 1A), and BMP2p-FITC peptides were synthesized based on the standard Fmoc-mediated solid-phase peptide synthesis strategy, with assistance from ChinaPeptides Co. Ltd. (Shanghai, China). Reserved-phase high-performance liquid chromatography (HPLC) was performed to purify the peptides on an Agilent system by using a Kromasil 100-5C18 column (5 μm, 4.6 mm × 250 mm, column temperature 25°C). The peptides were dissolved in dimethyl sulfoxide (DMSO) at 1 mg/10 μl for stocking. The phosphate-buffered saline solution (PBS, 0.02 mM, pH 7.2) was prepared in ultra-purified water (purified with a Merck Millipore pure water system to yield a minimum resistivity of 18.2 MΩ cm) and a purchased phosphate buffer salt (Beyotime Biotechnology, China). PEEK substrates were purchased from Weigao Group Medical Polymer Co., Ltd. (Shandong, China), and their diameter is 15.5 mm. Trypsin/EDTA solution (0.25%), streptomycin, and penicillin were purchased from Gibco BRL (United States). DMEM (Dulbecco’s modified eagle medium) and FBS (fetal bovine serum) were purchased from HyClone (United States). DBCO-Cy5 (#A130) was purchased from Click Chemistry Tools (United States). PEEK was first washed with ultrapure water, ethanol, and hydrogen peroxide/ammonia (1:1) three times, then dried, and treated with oxygen plasma. All other common biochemical reagents were used as received.

### Azide-DOPA_4_ or BMP2p Peptide Coating and Bio-Orthogonal BMP2p Grafting

After plasma treatment, the PEEK substrates were then immersed in the PBS solution of Azide-DOPA_4_ (0.01 mg/ml, pre-purged with nitrogen for 15 min) or BMP2p (0.1 mg/ml). After 12 h, the coated substrates were named Azide-DOPA_4_-PEEK and BMP2p-PEEK. Some Azide-DOPA_4_-PEEK substrates were further incubated with BMP2p (0.1 mg/ml) for another 12 h at room temperature and named as DOPA_4_@BMP2p-PEEK (Scheme 1A). All PEEK substrates were rinsed thoroughly using ultrapure water to wash away the unbounded peptides and then dried with nitrogen for further use. The PBS-immersed PEEK substrates were named PBS-PEEK and set as the control group.

### BMP2p Binding Efficiency on PEEK After Azide-DOPA_4_ Coating

The binding efficiency of Azide-DOPA_4_ coating on PEEK was examined by using a DBCO-Cy5 fluorescent dye. The PEEK substrates were first immersed in 0.01 mg/ml Azide-DOPA_4_ for 12 h at room temperature and then immersed in 10 μmol/L DBCO-Cy5 PBS solution for another 12 h. The samples were denoted as Cy5-labeled Azide-DOPA_4_-PEEK. To measure the bonding efficiency of BMP2p on PEEK, the Azide-DOPA_4_ coating PEEK substrates were then immersed in BMP2p-FITC PBS solution for 24 h to obtain Azide-DOPA_4_@BMP2p-FITC-PEEK. All PEEK substrates were rinsed using sterile deionized water three times before testing, and the fluorescence intensity was observed under a fluorescence microscope (Carl Zeiss, Germany).

### Characterization of the Surface of PEEK

Electrospray ionization mass spectrometry (ESI-MS) spectra were recorded on a Sciex API 150EX LC/MS with Agilent 1100 HPLC. Surface chemical compositions of the substrates were determined by using an X-ray photoelectron spectroscopy (XPS) instrument (ESCALAB MK II X-ray photoelectron spectrometer, VG Scientific). The static water contact angle (WCA) of PEEK at room temperature was evaluated with a KRÜSS DSA25 contact angle equipment (Germany). The surface morphology of the substrates was examined using atomic force microscopy (AFM, Dimension ICON, Bruker, United States). The morphologies of all samples were observed using a scanning electron microscope (SEM) (S-4800, Hitachi, Japan).

### Osteogenic Induction

Rat bone marrow–derived mesenchymal stem cells (rBMSCs) from the bone marrow of male rats were isolated and cultured in a growth medium (DMEM supplemented with 10% FBS, 100 U/ml of penicillin, and 100 μg/ml of streptomycin) as described in the previous study (Zhao et al., 2018; Huang et al., 2020). For osteogenic differentiation of rBMSCs, the cells (passage 3) were seeded on different surface-modified PEEK substrates and cultured in an osteogenic induction (OB) medium, which was prepared from a growth medium supplemented with 50 μg/ml of ascorbic acid and 10 mM of β-glycerol phosphate. In order to test the dose and osteogenic effects of BMP2p, rBMSCs were cultured in the OB medium complement with different doses of BMP2p (10/50/100/200 ng/ml) or 10 ng/ml recombinant human BMP-2 (rhBMP-2, PeproTech, United States); the medium was changed twice a week. All cells were cultured at 37°C with 5% CO_2_ atmosphere.

### Alkaline Phosphatase Staining and Activity Assay

To test the osteogenic activity, the ALP activity of rBMSCs cultured in the OB medium for 7 days was detected by ALP staining and ALP assay (24-well plate, 3×10^4^ cells per well). ALP staining of the cells was performed by using the BCIP/NBT ALP Color Development Kit (Beyotime Biotech, China) according to the manufacturer’s protocol. The images were acquired using a microscope (Carl Zeiss, Germany). In the ALP assay, the cells were washed with PBS 3 times and then lysed. ALP activity was determined colorimetrically using the ALP Assay Kit (Beyotime Biotech, China) and standardized on the total protein concentration calculated with the bicinchoninic acid (BCA) protein assay reagent (Beyotime Biotech, China).

### Alizarin Red Staining

Alizarin red staining was performed when rBMSCs were cultured in the OB medium for 2 weeks. The cells were fixed in 4% paraformaldehyde for 30 min at room temperature, rinsed with PBS 3 times each for 5 min, and then stained using Alizarin Red S (Solarbio, China), pH 4.2, for 30 min. The mineralized nodules containing calcium were stained as red spots and were photographed. The Alizarin red dye was subsequently extracted with 5% perchloric acid at room temperature for 20 min. Absorbance was then measured at 490 nm using a microplate spectrophotometer (BioTek, United States).

### Real-Time Quantitative RT-PCR

Messenger RNA (mRNA) of the cells was extracted according to the instructions of the TRIzol kit (Beyotime, China) after being cultured in the OB medium for 7 days. MRNA of the samples was reverse transcribed and qPCR was carried out (Bio-rad, United States). The relative gene expressions were calculated by the 2^−ΔΔCt^ method. GAPDH was selected to normalize the expression levels of the target genes. The results were presented in fold increase relative to the PBS-PEEK group. The primer sequences of the osteogenic-related genes, including ALP, Runt-related transcription factor 2 (Runx2), and type I collagen (Collagen I), are listed in Supplementary Table S1.

### Cytotoxicity Assay

The lactate dehydrogenase (LDH) activity assay was used in the cytotoxicity study. rBMSCs were cultured on different surface modified PEEK substrates for 3 days, the supernatant was collected, and the LDH content released from cultured rBMSCs was determined using an LDH assay kit (Beyotime Biotechnology, China) according to the manufacturer’s instructions. The LDH release from the PBS-pretreated PEEK group was set as 100%.

### Cell Adhesion

5 × 10^4^ rBMSCs were seeded on the different modified substrates placed in a 24-well cell plate with serum-free medium. After incubation at 37°C for 6 h, all the substrates were washed with PBS and fixed in 4% paraformaldehyde. After 30 min, the substrates were washed 3 times with PBS and incubated for 5 min with 0.4% Triton-X and 1 mM CaCl_2_ in PBS to punch the cell membrane at room temperature, and then stained with FITC-conjugated phalloidin (for staining F-actin stress fibers) and 4′-6-diamidino-2-phenylindole (DAPI, for staining nuclei) for 15 min. After staining, the substrates were washed three times with PBS and then examined under a fluorescence microscope.

### Foxp3+ Regulatory T Cell (Treg) Induction

All animal procedures were approved by the Soochow University Animal Care Committee and in accordance with the National Institute of Health’s Guide for the Care and Use of Laboratory Animals. C57BL/6 mice (male, 6–8 weeks) were used for T cell isolation. Mouse spleens were passed through a 70-μm nylon mesh to produce single-cell suspensions. CD4^+^ T cells were further magnetically enriched by negative selection according to the manufacturer’s instructions (Miltenyi Biotec, Germany), resulting in a purity ≥ 96%. Anti-CD3/CD28 antibodies were coated on a 24-well-plate to generate the activation plate, and isolated CD4^+^ spleen cells were cultured on the activation plate for 24 h to get the activating CD4^+^ spleen cells for further Treg-generated experiments (Supplementary Scheme S1A).

To assess the effects of Treg induction under different conditions, activated CD4^+^ spleen cells were cultured in a growth medium or stimulated with TGF-β (2 ng/ml) in the presence of IL-2 (20 units/ml) in AIM-V serum-free medium with or without BMP2p. Activated CD4^+^ spleen cells were also cultured in DOPA_4_@BMP2p–coated 24-well plate with or without stimulation with TGF-β and IL-2 (Supplementary Scheme S1B). After 24 h of stimulation, all the cells were collected, and the percentage of induced-Treg (iTreg, CD4^+^CD25 + Foxp3+) cells was analyzed by flow cytometry assay (FCA, Merck Millipore, Germany). As shown in Supplementary Scheme S1B, the culture supernatant from the Treg induction (T), DOPA_4_@BMP2p (DB), and Treg induction plus DOPA_4_@BMP2p groups (DB + T) were collected for further experiments.

### Osteogenic Effects of Conditional Medium From iTreg Cells

RBMSCs were cultured in the growth medium and OB medium with or without 50 ng/ml BMP2p, or different conditional mediums (70% OB medium with 30% supernatant). In order to test the osteogenic activity, ALP activity of rBMSCs cultured in a different medium for 7 days was detected by ALP staining. The positive area of ALP staining was analyzed through ImageJ, and five images for each sample and three samples for each group were chosen to perform the analysis.

### *In vivo* Rat Calvarial Bone Defect Model

A total of 18 healthy male SD (Sprague–Dawley) rats, weighing around 300 g, were used to construct the 5-mm critical calvarial bone defect model. In brief, all the rats were anesthetized by intraperitoneal injection of 2% sodium pentobarbital, and a 5-mm electric drill was used to drill holes on both sides of the calvarial suture to remove the entire calvarial bone. The PEEK substrates with a differently modified surface were randomly placed in the defect holes, and the holes without the implant were set as the control group. Then, the muscles and skin were sequentially sutured with absorbable 4-0 surgical sutures. After the operation, the rats were reared in separate cages, and penicillin (150,000 Units) was injected intramuscularly once a day after the operation for 3 days. Eight weeks after the surgery, all rats were killed by an overdose of anesthesia and calvarial bones were harvested.

### Micro-CT Analysis and Histological Staining

The bone samples were fixed with neutral formalin for 48 h and then micro-computed tomography (SkyScan 1176, SkyScan, Belgium) scanning was performed. The parameters are as follows: 65 kV, 385 mA, and 1-mm aluminum filter. Three-dimensional images were reconstructed by using Mimics, and new bone volume/tissue volume (BV/TV) and bone mineral density (BMD) of the defect area were analyzed by CTAn. All bone samples were embedded in neutral resin and then were sliced with a hard tissue microtome according to the previously reported method (Zhao et al., 2018). The sections were stained with hematoxylin and eosin (H&E and Masson). The images were acquired by using a microscope (Carl Zeiss, Germany), and the bone-to-implant contact ratio (BIC, %) was calculated as the percentage of PEEK’s circumference that was in direct contact with bone mineral in histological sections.

### Statistical Analysis

All quantitative data were expressed as mean ± standard deviation (*S.D.*) with no less than three replicates for each experimental condition. Statistical differences between two groups were analyzed by Student’s t-test, and significant differences between more than two groups were analyzed by one-way analysis of variance (ANOVA), followed by Tukey’s post hoc test. Differences between the two groups were considered statistically significant when the *p*-value was less than 0.05.

## Results

### Peptide Synthesis

The mussel-inspired DOPA_4_ peptide was prepared according to previously reported methods via a standard Fmoc-mediated solid-phase synthesis strategy (Pan et al., 2016; Liu et al., 2018; Ma et al., 2019). To form mussel-like molecular binding, acetonide and Fmoc-protected DOPA (Fmoc-DOPA (acetone)-OH) was used for peptide synthesis. The amino acid sequence of this peptide was primarily designed with tetravalent DOPA units. To facilitate the molecular twist of the multiple catecholic groups and enhance the mussel-like surface binding, the DOPA units were further improved by alternately inserting two glycine (G) and one lysine (K) molecule as spacers. In this case, the amino group of the K spacer could also be functionalized with carboxylated and azide-bearing polyethylene glycol (PEG), finally leading to the Azide-DOPA_4_ peptide (Ac-(DOPA)-G-(DOPA)-K(PEG5-Azido)-(DOPA)-G-(DOPA)) (N→C) (Scheme 1A). BMP-2 functional peptides were conjugated with DBCO by N-hydroxysuccinimide and maleimidethiol coupling (Scheme 1A).

After being purified by HPLC (Supplementary Figures S1A,B), ESI-MS was then used to characterize the synthesized peptides according to the measured molecular weight. The monoisotopic mass [M + H]^+^ of Azide-DOPA_4_ and [M+2H]^2+^ of BMP2p were measured at 1,336.79 and 1,439.8 Da, respectively, consisting with their theoretical molecular weight 1,336.4 and 2,877.3, respectively (Supplementary Figures S1C,D). These results confirmed the successful synthesis of the azide-capped mussel-inspired peptide (Azide-DOPA_4_) and DBCO-conjugated BMP-2 functional peptides (BMP2p).

### Peptide Coating and Surface Characteristics

**FIGURE 1:**
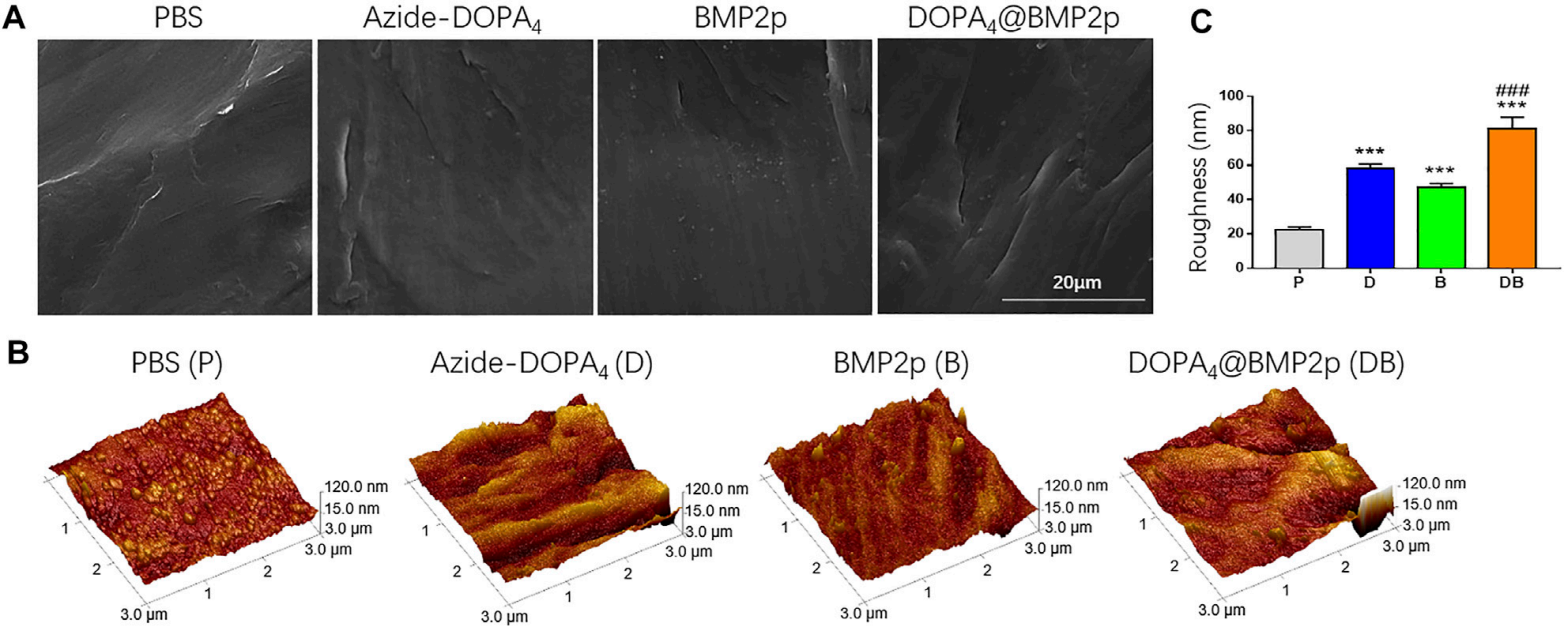
Surface morphology of PEEK after different modifications. **(A)** SEM images of the PEEK substrates with different treatment methods, **(B,C)** AFM images and the surface roughness measure of the PEEK substrates after different treatment methods. Statistically significant differences are indicated by ***p < 0.001 compared with the PBS-PEEK group and ^###^ *p* < 0.001 compared with the BMP2p group. Abbreviations: P (PBS), D (Azide-DOPA_4_), B (BMP2p), and DB (DOPA_4_@BMP2p).

**FIGURE 2:**
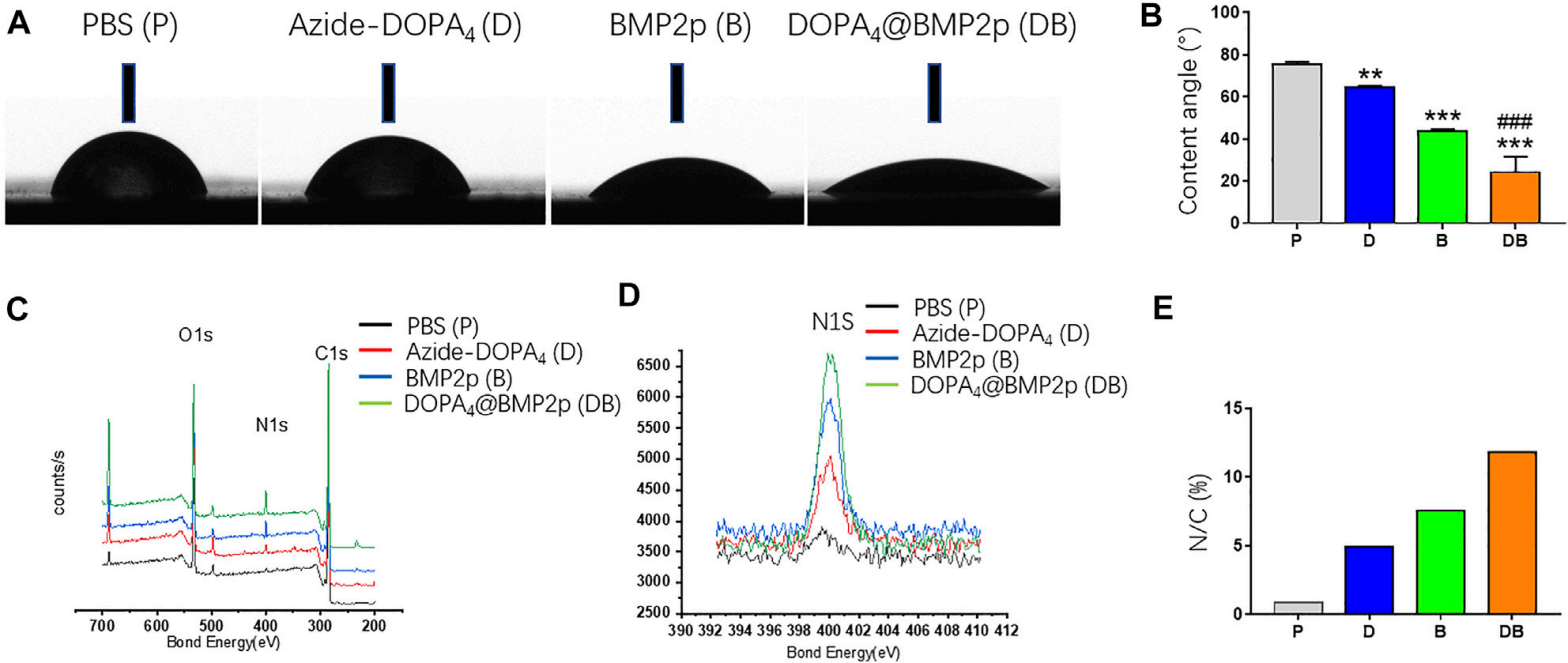
Surface characterization of PEEK after different modifications. **(A)** Images of corresponding water contact angles. **(B)** Quantitative results of water contact angles. **(C–E)** XPS spectra and the surface N/C elemental ratios of PEEK substrates after different modifications. Statistically significant differences are indicated by **p < 0.01 or ***p < 0.001 compared with the PBS-PEEK group and ^###^ *p* < 0.001 compared with the BMP2p group. Abbreviations: P (PBS), D (Azide-DOPA_4_), B (BMP2p), and DB (DOPA_4_@BMP2p).

In order to study the surface characteristics after different peptide coatings on PEEK, we first observed the surface changes of materials by SEM, and no apparent differences could be found (Figure 1A). AFM was then employed to characterize the surface topography. Compared with the PBS-PEEK group, the roughness of the Azide-DOPA_4_ and BMP2p groups was significantly increased, while there were many nano-sized embosses unevenly deposited on the DOPA_4_@BMP2p group surface, and the roughness measure was also the highest (Figures 1B,C). Furthermore, WCA test results demonstrated that the surface of PBS-PEEK was strongly hydrophobic, and the surface wettability of the substrates showed significant improvement after peptide coating (Figure 2A). According to the calculation of the corresponding WCA profile on the surface of the material, we found that the DOPA_4_@BMP2p-PEEK material had the best hydrophilicity (Figure 2B). Therefore, these results primarily indicated the feasibility of our DOPA_4_ peptide combined with BMP2p for surface modification of PEEK.

Following that, we used XPS to confirm the changes of surface elemental compositions of PEEK after different surface modifications. Due to the high nitrogen (N) content of Azide-DOPA_4_ and BMP2p, all peptide-treated substrates showed a remarkably enhanced N1s signal (400.12 eV) compared with that of the PBS-PEEK group, which suggested that the peptide was successfully loaded onto the surface of PEEK. Compared with the BMP2p-PEEK group, DOPA_4_@BMP2p-PEEK showed a higher N1s signal. Since this peak corresponds to the amide in the peptide bond, it reminds us that Azide-DOPA_4_ coating can significantly increase the binding of BMP2p on the surface of the PEEK (Figures 2C,D). The quantitative results further revealed that the N/C atomic ratios significantly increased in all the peptide-treated groups, especially in the DOPA_4_@BMP2p-PEEK group (Figure 2E).

**FIGURE 3:**
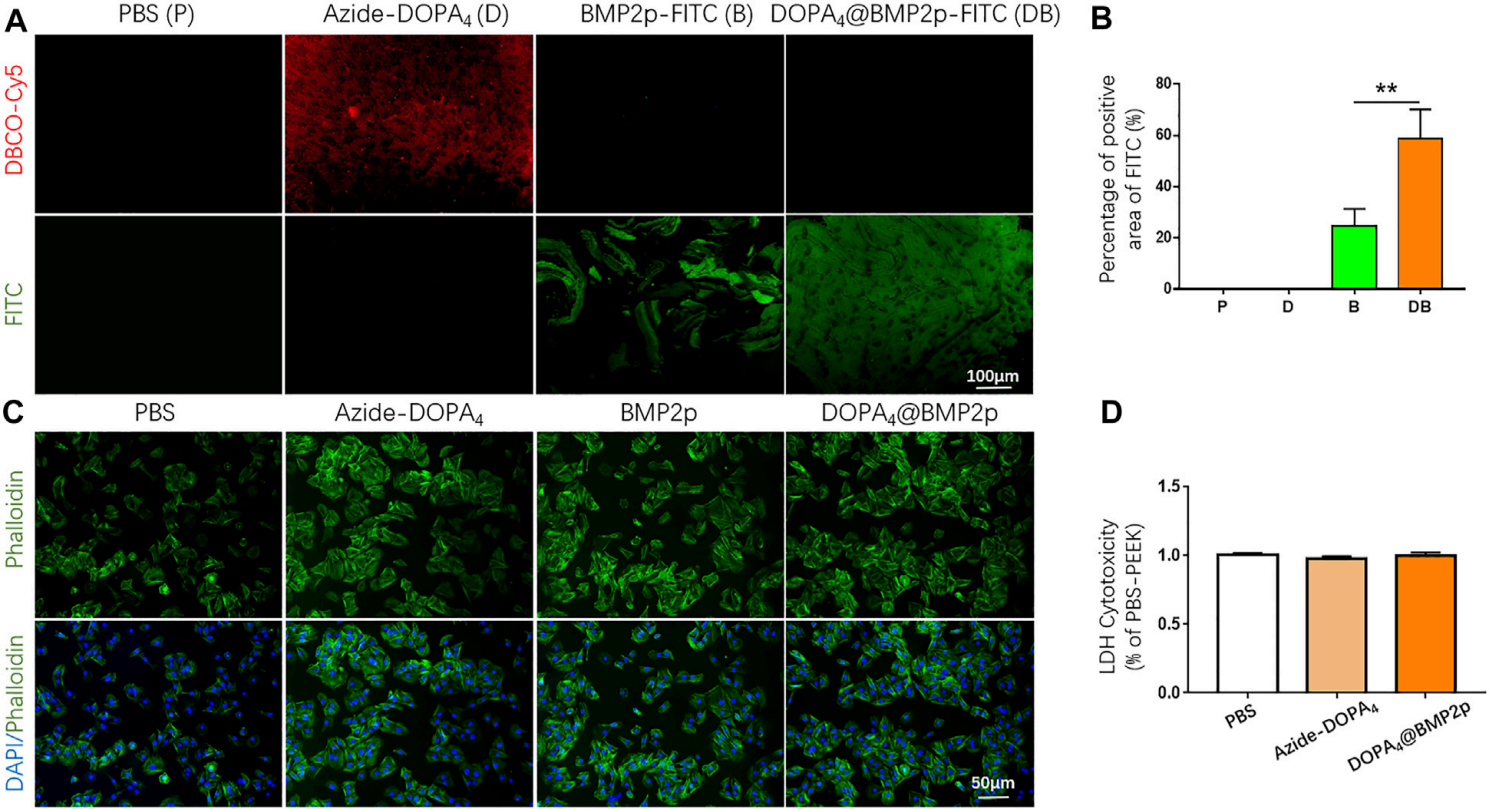
Biocompatibility of different modified substrates, and bioclickable BMP2p binding on PEEK after Azide-DOPA_4_ coating. **(A)** Fluorescence images of PEEK substrates treated with fluorescent dyes labeled Azide-DOPA_4_ and BMP2p-FITC. **(B)** Percentage of FITC positive areas of PEEK after different treatment methods, **(C)** Morphologies of adherent rBMSCs after 6 h of culture. The cells were stained with DAPI (nuclei, blue) and Alexa Fluor 488–conjugated phalloidin (actin filaments, green). **(D)** Quantification of LDH. Abbreviations: P (PBS), D (Azide-DOPA_4_), B (BMP2p-FITC), and DB (DOPA_4_@BMP2p-FITC).

In order to detect the coating condition of Azide-DOPA_4_ on PEEK, we used DBCO-Cy5 fluorescent dye to label Azide-DOPA_4_ on PEEK through a bio-orthogonal reaction. As observed in the images given in Figure 3A, red fluorescence can be observed on the surface of DBCO-Cy5-Azide-DOPA_4_-PEEK (group D) and this confirmed the successful embedding of Azide-DOPA_4_ on PEEK. We also synthesized FITC-labeled BMP2p (BMP2p-FITC) to further confirm the efficiency of BMP2p conjugating on PEEK, especially after Azide-DOPA_4_ coating. As we expected, specific fluorescence can be observed on the surface of DBCO-Cy5-Azide-DOPA_4_-PEEK (group D) and BMP2p-FITC-PEEK (group B) under the fluorescence microscope after 12 h of immersion at room temperature. Scattered green fluorescence can be seen on the surface of the group B material, while uniformly distributed green fluorescence can be seen on the surface of the DOPA_4_@BMP2p-FITC-PEEK (group DB) material (Figure 3A). The FITC fluorescence positive area was significantly increased in group DB compared to group B, which further verified that the PEEK material could bind more with BMP2p after Azide-DOPA_4_ surface coating (Figure 3B).

We further performed a cell adhesion experiment of rBMSCs cultured on the different modified substrates, and the effects of cell adhesion were investigated by F-actin cytoskeleton staining (phalloidin, green). As shown in Figure 3C, rBMSCs could adhere on all the substrates after 6 h of culture, while the organization of F-actin networks for cells adhered on the group of DOPA_4_@BMP2p exhibited larger amount and improved spreading shape compared with other three groups. However, there was also an enhancement of rBMSC adhesion in the Azide-DOPA_4_ and BMP2p-modified groups compared with the PBS group, which is consistent with the surface characterization results of different modified PEEK substrates. Besides, the interaction between BMP-2 and BMPRs could also enhance cell adhesion. LDH assay provides a simple and reliable method for determining cellular cytotoxicity, especially in finding the damage inflicted on the plasma membrane. We assessed the biocompatibility of different modified PEEK surfaces via LDH assay after we synthesized the peptides. As shown in Figure 3D, there was no statistical difference of LDH content released from cells cultured on peptide-grafted substrates compared to the PBS-pretreated PEEK substrates, indicating that peptide modification of PEEK displayed no cytotoxicity. Through the abovementioned analysis of the surface characteristics of the material, we have confirmed that the DOPA_4_@BMP2p surface modification significantly improves the characteristics of PEEK. The surface roughness was obviously increased and the hydrophilicity was improved after coating, which is better for cell adhesion on PEEK. Besides, PEEK can bind more with BMP2p after Azide-DOPA_4_ coating by bioclickable conjugation. More importantly, compared with other physical and chemical methods, this combination method is convenient and stable.

### Osteogenesis Induction Capability of PEEK Materials With DOPA_4_-BMP2p Modified Surface *in vitro*

In order to test the dose and osteogenic effects of our BMP2p, rBMSCs were cultured in the OB medium with different doses of BMP2p (10/50/100/200 ng/ml) or 10 ng/ml rhBMP-2, and the cells cultured in the OB medium were set as the control. We first performed ALP staining after 7 days of cell culture, which is an early marker for evaluating the metabolic activity of osteoblasts. Compared with the control group, the number of ALP staining positive cells increased significantly when cultured with exogenous 10 ng/ml rhBMP-2 or 100 ng/ml BMP2p (Supplementary Figure S1A), and the results of ALP activity were consistent with those of the staining (Supplementary Figure S1C). Matrix mineralization is an indicator in the later stage of osteogenesis, which is also an important index of enhanced osteogenesis. We further performed Alizarin red staining to observe its effect on cell mineralization after 14 days of culture, and the mineralization of rBMSCs was significantly enhanced when cultured with exogenous 50/100/200 ng/ml BMP2p (Supplementary Figures S1B,D). From the results mentioned above, we identified the osteogenic activity of our BMP2p, and 100 ng/ml BMP2p even displayed better osteogenic promoting effects than 10 ng/ml rhBMP-2 (Supplementary Figure S1D).

**FIGURE 4:**
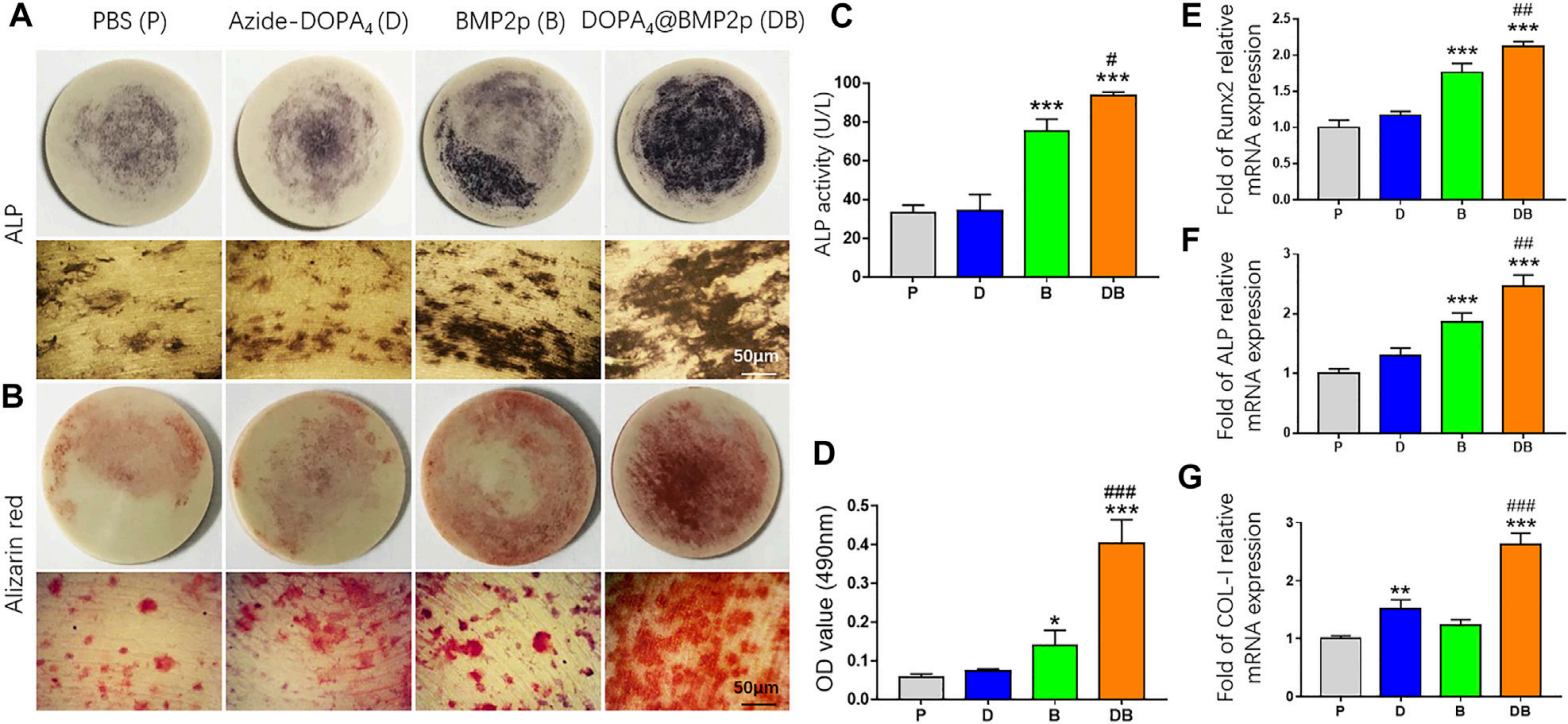
*In vitro* osteogenesis induction capability of PEEK after different surface modifications. **(A)** Representative images of ALP staining of rBMSCs on different PEEK substrates after 7 days of culture in the osteogenic induction medium (upper: general photo of whole PEEK and lower: images under the microscope). **(B)** Representative images of Alizarin red S staining after 14 days of osteogenic differentiation (upper: general photo of whole PEEK and lower: images under the microscope). **(C,D)** Quantitative analysis of ALP activity and Alizarin red–stained mineral layer. **(E–G)** QPCR analysis of osteogenic differentiation genes of rBMSCs on different PEEK substrates after 5 days of culture in the osteogenic induction medium. Statistically significant differences are indicated by *p < 0.05, **p < 0.01, or ***p < 0.001 compared with the PBS-PEEK group (P) and ^#^ *p* < 0.05 and ^###^ *p* < 0.001 compared with the BMP2p-PEEK group (B). Abbreviations: P (PBS), D (Azide-DOPA_4_), B (BMP2p), and DB (DOPA_4_@BMP2p).

We then characterized osteogenic differentiation of rBMSCs on different surface-modified PEEK substrates by testing the activity of ALP. After being cultured in the OB medium for 7 days, ALP staining positive cells can be found in all PEEK groups. There were more ALP-positive cells in the BMP2p and DOPA_4_@BMP2p groups (Figure 4A). By normalizing the ALP protein contents, the BMP2p-PEEK group showed 2.26-fold higher ALP activity, and the DOPA_4_@BMP2p group showed 2.81-fold higher ALP activity than the PBS group. More importantly, the DOPA_4_@BMP2p group had significantly higher ALP activity than that of the BMP2p group (Figure 4C). This result indicated early enhancement of osteogenicity. We further stained the rBMSCs with Alizarin red after 14 days of culture in the OB medium, and positive staining can also be found in all PEEK groups (Figure 4B). The quantitative analysis results of Alizarin red were consistent with those of the ALP. The BMP2p-PEEK group showed 2.43-fold higher Alizarin red content, and the DOPA_4_@BMP2p group showed 6.95-fold higher Alizarin red content than the PBS group. Moreover, the DOPA_4_@BMP2p group showed 2.86-fold higher mineralization effects than the BMP2p group (Figure 4D). Then, we examined the expression of the osteogenic-related genes of rBMSCs cultured on different PEEK materials. Compared with the PBS-PEEK, gene expression level of Runx2, ALP and COL-I were also significantly upregulated in the DOPA_4_@BMP2p group after 7 days of osteogenic induction (Figures 4E–G). Collectively, the osteogenetic capability of PEEK is significantly enhanced in the DOPA_4_@BMP2p group through bioclickable conjugation.

### Bone Defect Repairing Effects of PEEK Materials With the DOPA_4_@BMP2p Modified Surface *in vivo*

**FIGURE 5:**
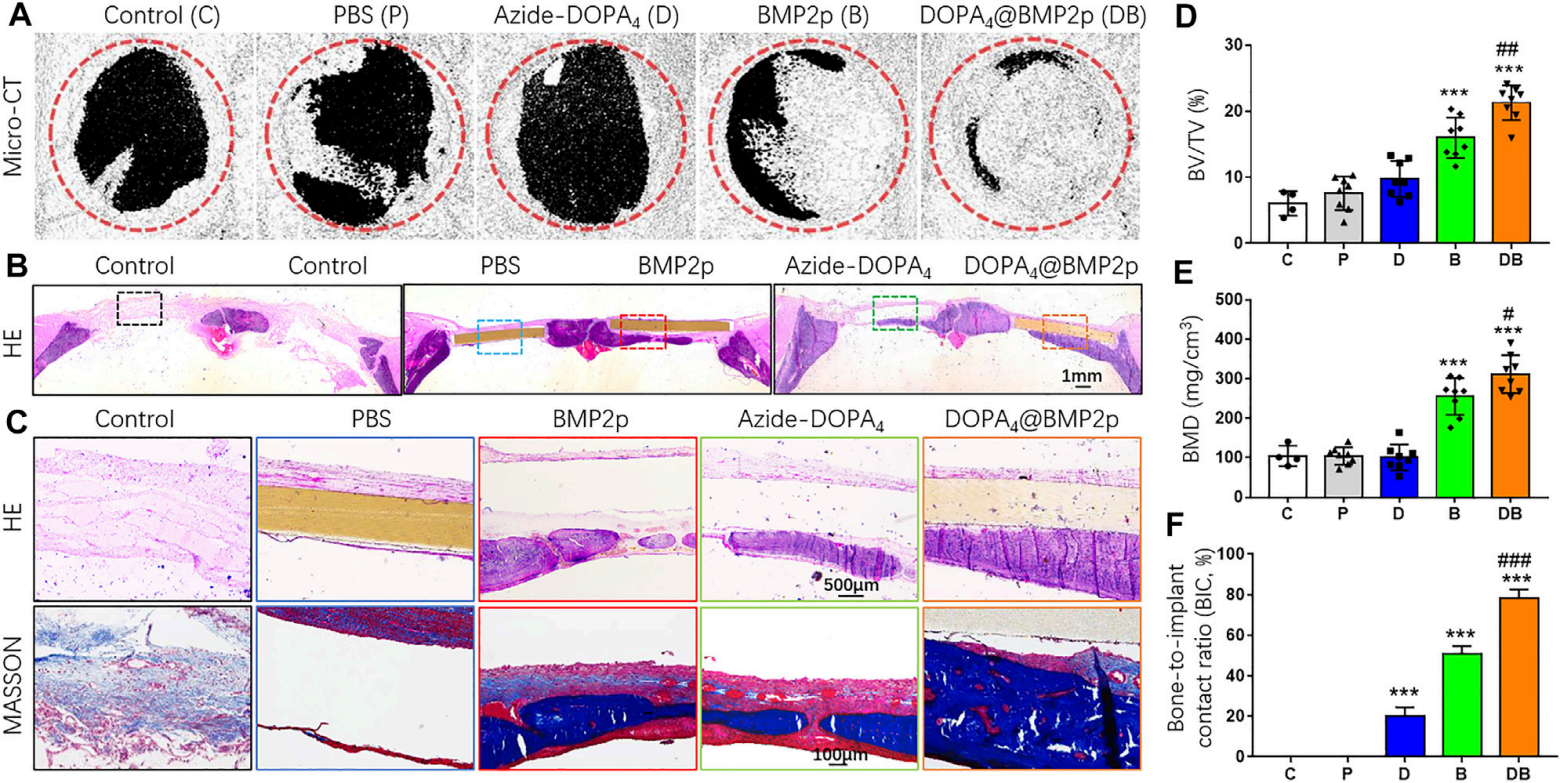
*In vivo* bone defect repairing effects of PEEK after different surface modifications. **(A)** Micro-CT 3D-reconstructed images and **(D,E)** quantitatively evaluating the peri-implant bone generation according to the percentage bone volume (BV) among tissue volume (TV) (BV/TV) and bone mineral density (BMD). **(B)** H&E histological section staining images of the *in vivo* osseointegration of different surface-modified PEEK implants at low magnification and **(C)** high magnification. **(D)** Masson’s staining images of the different surface-modified PEEK implants. **(G)** Histomorphometric analysis of bone-implant contact (BIC) percentage of different surface-modified PEEK implants. Data are presented as the mean ± SD, *n* = 4–8. Statistically significant differences are indicated by ***p < 0.001 compared with the sham control group (group C) and ^#^ *p* < 0.05, ^##^ *p* < 0.01, or ^###^ *p* < 0.001 compared with the BMP2p-PEEK group (group B). Abbreviations: C (control), P (PBS), D (Azide-DOPA_4_), B (BMP2p), and DB (DOPA_4_@BMP2p).

In order to examine the osteogenic induction of PEEK after different surface modifications *in vivo*, we constructed a rat model with the 5-mm critical calvarial bone defects and implanted them with surface-modified PEEK. The rat calvarial bone samples were collected 8 weeks after the surgery. Micro-CT scanning and histological staining of hard tissue sections were used to determine the bone defect repairing effect of different surface-modified PEEK materials. Three-dimensional reconstruction images of Micro-CT scanning showed that there was no obvious new bone formation in the bone defect area of the control group (C). Compared with group C, there were several scattered new bone formations in the PBS-PEEK (P) group, and some new bone formations around the edge of the bone defect area were found in the Azide-DOPA_4_-PEEK (D) group. Both the BMP2p-PEEK group (B) and DOPA_4_@BMP2p-PEEK (DB) group showed apparent new bone formation, and the group DB grew more new bones, which almost covered the bone defect area (Figure 5A). We further analyzed the new bone volume (BV/TV) and bone mineral density (BMD) of the defect area. Compared with group C, the BV/TV and BMD in groups P and D were slightly increased, but the difference had no significance, while in groups B (2.65-fold) and DB (3.53-fold), they were significantly increased. BMD results showed no difference in groups P and D compared with group C, that is, there was a 2.44-fold increase in group B and a 2.97-fold increase in group DB. Compared with group B, the BV/TV and BMD were significantly higher in group DB (Figures 5D,E).

Furthermore, we performed H&E and Masson’s staining of the hard tissue slices of the calvarial bone samples. No foreign body giant cells or fibrous capsules were found around the bone-implant interface in each surface-modified PEEK group. There was no apparent new bone formation in the bone defect area in groups C and P; a small amount of new bone formation was found under the PEEK material in the group D, and some new bone and new blood vessels were seen in group B, but the bonding interface between the PEEK material and the new bone was not good. In group DB, a large number of new bones were continuously distributed along the bottom of PEEK (Figures 5B,C). The bone-to-implant contact ratio (BIC, %) was calculated, and group DB had the best bone-to-implant contact (Figure 5F). These *in vivo* data indicated that, compared with the single-peptide surface modification, Azide-DOPA_4_ combined with BMP2p surface modification could effectively enhance the osteogenicity and osseointegration ability of PEEK. To the best of our knowledge, our study is the first one to improve the osseointegration and osteoinduction capacity of PEEK through the DBCO-Azide copper-free click method.

### Synergistic Osteogenesis Effect of DOPA_4_@BMP2p With Anti-Inflammation of iTreg

**FIGURE 6:**
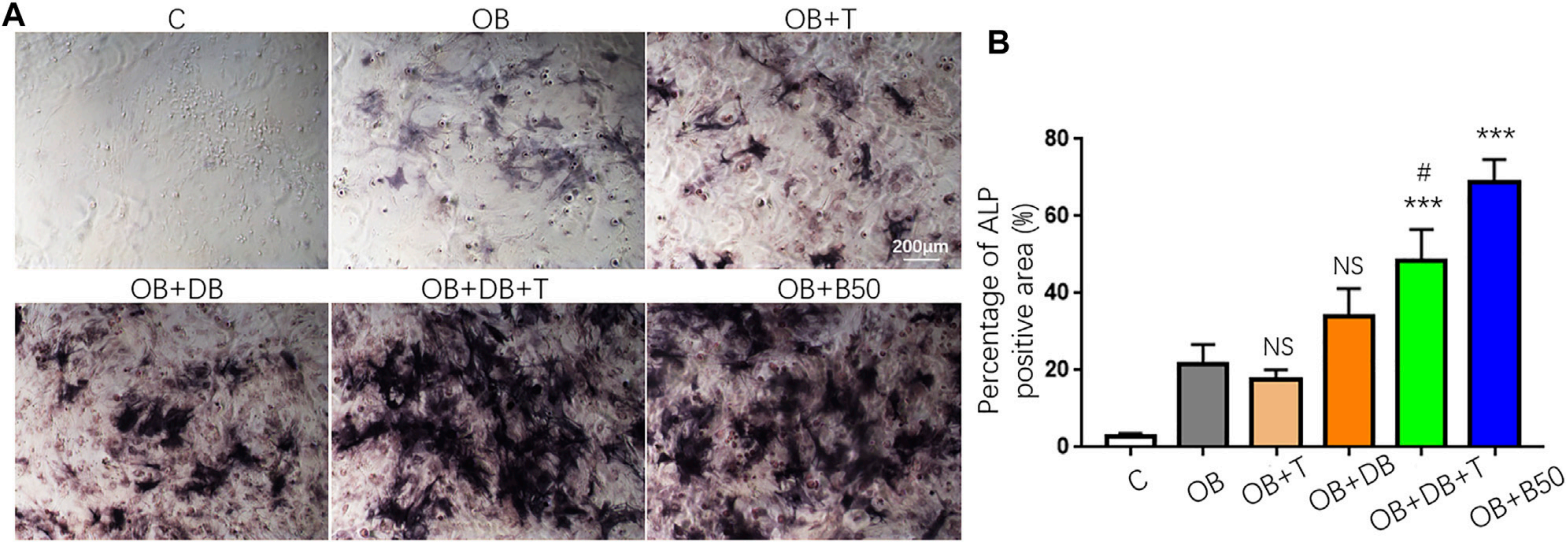
Synergistic osteogenesis effect of DOPA_4_@BMP2p coating and Treg cells. **(A)** Representative images of ALP staining of rBMSCs cultured in a different medium. **(B)** Percentage of ALP staining positive area (%). Data are presented as the mean ± SD, *n* = 4. Statistically significant differences are indicated by ***p < 0.001 compared with the OB group (OB), and NS means not statistically significant. ^#^ *p* < 0.05 compared with the OB + DB group. Abbreviations: C (control), OB (osteogenic induction medium), OB + T (conditional medium consist of OB medium and supernatant from Treg induction group), OB + DB (conditional medium consist of OB medium and supernatant from DOPA_4_@BMP2p group), OB + DB + T (conditional medium consist of OB medium and supernatant from Treg induction + DOPA_4_@BMP2p group), and OB + B (OB medium plus 50 ng/ml BMP2p).

Numerous studies have implicated the immunomodulatory properties of BMP-2, which also plays an indispensable role in bone regeneration (Shu et al., 2018; Wei et al., 2018; Pajarinen et al., 2019). Consequently, we examined whether our BMP2p also retains these immune regulatory effects. CD4+Foxp3-conventional T cells could be induced to Foxp3+ Treg (iTreg) cells in the presence of TGF-β and IL-2 *in vitro* (Joetham et al., 2020). As shown in Supplementary Scheme S1, we cultured activated CD4^+^ spleen cells under different conditions and collected the supernatant. The number of iTreg cells (CD4^+^CD25 + Foxp3+) was determined by flow cytometry. Unexpectedly, the exogenous addition of 50 ng/ml BMP2p or DOPA_4_@BMP2p coating did not promote the generation of iTreg cells (Supplementary Figure S3). However, the conditional medium from DOPA_4_@BMP2p coating iTreg cells (OB + DB + T) showed better osteogenic activity than DOPA_4_@BMP2p coating activated CD4^+^ T cells (OB + DB) and classical iTreg cells (OB + T). These results suggested that DOPA_4_@BMP2p coating may have a synergistic effect with iTreg cells to promote osteogenesis (Figure 6).

## Discussion

It is believed that the surface characteristics of the material (the composition of the surface, the roughness morphology, and the hydrophilicity) are very essential for its osseointegration, and the medium roughness and strong hydrophilic surface is more conducive to cell activity and osseointegration (Stach and Kohles, 2003; Feller et al., 2014). Compared with traditional metallic bone tissue implantation materials, such as stainless steel, titanium, and its alloys, PEEK has excellent properties, such as stable chemical structure, good biocompatibility, wear-resistance, and transmittable X-rays. PEEK also has an elastic modulus closer to the cortical bone, which can effectively reduce or eliminate the stress-shielding effect, thereby reducing or avoiding bone resorption (Kurtz and Devine, 2007). However, surface hydrophobicity, lack of bone conduction, and osseointegration capabilities limit its further clinical application. Over the past decades, surface modification has been widely used to improve its biological activity and osteogenic properties (Ouyang et al., 2016; Chen et al., 2017; Wang et al., 2019). Nonetheless, these methods mostly involve tedious chemical reactions and complicated surface treatments, which may also compromise the controllability, operability, and reproducibility of a multicomponent bioactive surface.

Like Mfps, our mussel-inspired peptide (DOPA) could stably bind to the Ti implants via a facile self-organized multivalent coordinative interaction (Guo et al., 2019), and further improved the osseointegration of Ti screws in osteoporotic conditions by combining with two bioactive peptides (cell adhesive peptide RGD and osteogenic growth peptide OGP) (Zhao et al., 2018). Here, we developed a biomimetic method for introducing an osteogenic bioactive coating onto PEEK surfaces by combining bio-orthogonal conjugation with mussel-inspired adhesive chemistry. A mussel-inspired peptide with a clickable azide group (Azide-DOPA_4_) was synthesized for the first-step grafting via mussel adhesion mechanism, and the osteogenic moiety BMP2p spontaneously binds onto the mussel-inspired peptide layer through bio-orthogonal click chemistry. According to the analysis of the surface characteristics of the DOPA_4_@BMP2p-modified PEEK material, we confirmed that the surface roughness was obviously increased, and the hydrophilicity was improved after coating, which is better for cell adhesion on PEEK. Compared to traditional chemical methods, the combination of mussel adhesion and bio-orthogonal chemistry features simplicity, rapidness, and high efficiency.

BMP is a member of the TGF-β (transforming growth factor-β) superfamily and plays an influential regulatory role in embryogenesis, skeletal growth, remodeling, and repair. A large number of studies have shown that BMP-2 can increase the expression of osteoblast functional proteins, enhance the activity of ALP, and promote the osteogenic-related cells to form mineralized nodules *in vitro*. Besides, rhBMP-2 is currently available for orthopedic usage (Khan and Lane, 2004). However, rhBMP-2 still has some limitations, such as easy decomposition, short half-life, and high cost. BMP-2 is also widely used in biomaterial tissue engineering (Liu et al., 2007). Nevertheless, the material-loaded BMP-2 may be released in large quantities in a short period of time, which has certain risks, such as swelling, seroma, and even an increased risk of cancer (Carreira et al., 2014). The polypeptide is a chain of more than 20 and less than 50 amino acids bound together via covalent peptide bonds. Polypeptides can be synthesized using various chemical means, and the cost is low. Moreover, compared with high molecular proteins, the biological function of the polypeptide is more stable and durable. In our study, Azide-DOPA_4_ and BMP2p can be easily and safely combined on PEEK. Moreover, compared with immersion in the BMP2p-FITC solution, we verified that PEEK material could bind more with BMP2p after Azide-DOPA_4_ surface coating through bio-orthogonal chemistry. Likewise, the osteogenesis induction capability of DOPA_4_@BMP2p PEEK is significantly enhanced *in vitro*. Interestingly, we also found the synergistic effect of Azide-DOPA_4_ and BMP2p on osteogenesis *in vivo*, which also appeared in other materials with dual or multiple biological activity or surface modification materials (Naskar et al., 2017; Yang et al., 2020b). The Azide-DOPA_4_ peptide might provide a site for cells to attach to the matrix, thereby enhancing the interaction between the BMP2p and transmembrane protein receptors of the cell and further increasing bone formation. Besides, the potential immune regulatory functions of BMP2p may also play an important role in this synergistic osteogenesis effect.

Immune-bone crosstalk is thought to play a crucial role in implant integration in bone tissue. After trauma caused by the surgical implant procedure (Anderson et al., 2008), the immune response runs not only in parallel but also resulting in a complex network of reactions that dictate the long-term fate of the implant (Jones, 2008). Many types of immune cells play a notable role in such a process, including monocyte–macrophage cells, lymphocytes (T, B cells), and NK cells (Smith et al., 2009). The critical role of macrophages in the inflammatory balance has been well established. They can polarize two main different phenotypes that depend on local conditions, while the classical (M1) phenotype is favorable for proinflammation and alternative (M2) for anti-inflammation and tissue regeneration (Gu et al., 2017). Lymphocytes interact with macrophages and bone cells, thus eliciting their participation in the osseointegration process (Chen et al., 2014).

It is becoming increasingly clear that a balanced immune response is an essential condition for successful bone regeneration (Mountziaris et al., 2011). CD4^+^CD25 + Foxp3+ Treg cells play a crucial role in the maintenance of immune and bone homeostasis. Bone homeostasis is mostly mediated by the interaction between osteoblastic bone formation and osteoblastic bone resorption. The protective role of Treg cells in bone loss is mainly by inhibiting the formation of osteoclasts *in vitro* and *in vivo* (Zaiss et al., 2007; Zaiss et al., 2010a). For example, Treg cells inhibit the osteoclasts’ differentiation through paracrine signaling of TGF-β and IL-4 *in vitro* (Kim et al., 2007). Treg cells also protect TNF-α–induced bone destruction and ovariectomy-induced bone loss *in vivo* (Zaiss et al., 2010a; Zaiss et al., 2010b). The interplay between Treg cells and osteoblasts has not been completely understood. It has been demonstrated that Treg cells may directly promote osteoblast differentiation from progenitors (MSC) by inhibiting CD4^+^ conventional T-cells and decrease their secretion of IFN-γ and TNF-α (Liu et al., 2011; Liu et al., 2015). On the other hand, Treg cells have also been implicated in promoting the differentiation of osteoblasts directly (Lei et al., 2015). Research on intermittent PTH–induced bone anabolism found that Treg cells are involved in the upregulation of the expression of wnt10b, an osteogenic factor secreted by the CD8^+^ T cells, which was also demonstrated in the stimulation of bone formation by oral supplementation with *Lactobacillus rhamnosus* GG (LGG) (Li et al., 2014; Tyagi et al., 2018). Additionally, bone healing and repair also can be promoted by Treg cells by enhancing bone formation and suppressing osteoclastic bone resorption (Fischer et al., 2019). More and more research studies suggest immune modulation as a novel therapeutic strategy to enhance implant osseointegration.

Recently, several studies found the immunomodulatory properties of BMP-2 to manipulate the osteoimmune environment for favorable bone regeneration (Shu et al., 2018). BMP-2 can increase the recruitment and migration of macrophages *in vitro* and increased the infiltration of macrophage populations of M2 phenotypes in the subcutaneous implants (Wei et al., 2018). The addition of BMP-2 also significantly increases the ability of TGF-β to promote the generation of Foxp3+ induced Treg cells (Lu et al., 2010). In our study, we did not find apparent promotion of the generation of iTreg cells by exogenous addition of 50 ng/ml BMP2p or DOPA_4_@BMP2p coating. We speculated that one of the possible reasons is that the spatial structure of rhBMP2 and BMP2p is different, and the function and effect on the generation of iTreg cells of BMP2p may decrease or even be lost. On the other hand, the dosage of BMP2p we used may not be enough. However, the conditional mediums from DOPA_4_@BMP2p coating–induced Treg cells showed better osteogenic activity than DOPA_4_@BMP2p coating–activated CD4^+^ T cells and classical-induced Treg cells. This suggested that DOPA_4_@BMP2p coating may have a synergistic effect with iTreg cells to promote osteogenesis, which may also be the reason for the synergistic effect of Azide-DOPA_4_ and BMP2p on the osteogenesis we found *in vivo*. The PEEK material cannot be decalcified, and we do not have a good way to carry out immunofluorescence staining on the tissue embedded in the neutral resin to further verify the effects of Treg cells in the bone defect area *in vivo*. The effects and mechanism of the synergistic effect of DOPA_4_@BMP2p coating material with Treg cells *in vivo* still need to be further explored in future.

## Conclusion

In summary, we reported here an improved mussel-inspired surface engineering strategy for PEEK by the combination of mussel-inspired peptide and bio-orthogonal click chemistry. The main idea of this improved strategy is to synthesize an Azide-bearing mussel-inspired peptide. With the Azide residues on the DOPA_4_-modified surface, this strategy enables a second-step bio-orthogonal conjugation of DBCO-capping BMP2p via DBCO-Azide clicking. *In vivo* results demonstrated that our Azide-DOPA_4_ combined with the BMP2p surface modification method can increase new bone formation around the PEEK implant and also significantly improve the integration ability of PEEK, which will reduce the possibility of implant loosening after surgery. Importantly, we also found that DOPA_4_@BMP2p coating has a synergistic effect with induced Foxp3+ regulatory T (iTreg) cells to promote osteogenesis. This research provides a theoretical and experimental basis for the further application of PEEK materials in trauma and tissue engineering scaffolds. In addition, the molecular specificity of bio-orthogonal conjugation and the universality of the mussel adhesion mechanism reflected in our strategy may provide a versatile surface bioengineering method for a broader range of biomedical implants.

## Data Availability

The original contributions presented in the study are included in the article/Supplementary Material; further inquiries can be directed to the corresponding authors.
