# Supplementary material for: Bioclickable Mussel-Derived Peptides With Immunoregulation for Osseointegration of PEEK
Source: Front Bioeng Biotechnol. 2021 Nov 24;9:780609. doi: 10.3389/fbioe.2021.780609 (PMC8652040; doi:10.3389/fbioe.2021.780609)
Supplement: Supplementary file 1 [file DataSheet1.docx]

**Supplementary data**

**Table S1.** Primers of genes for qPCR

| Gene | Primer sequence（5’→3’） |
| --- | --- |
| Runx2 | F:5’-CCAACTTCCTGTGCTCCGTG-3’ |
|  | R:5’-GTGAAACTCTTGCCTCGTCCG-3’ |
| ALP | F:5’-AACGTGGCCAAGAACATCATCA-3’ |
|  | R:5’-TGTCCATCTCCAGCCGTGTC-3’ |
| Collagen I | F:5’-TGGATGGCTGCACGAGT-3’ |
|  | R:5’-TTGGGATGGAGGGAGTTTA-3’ |
| GAPDH | F:5’-GCAAGTTCAACGGCACAG-3’ |
|  | R:5’-CGCCAGTAGACTCCACGAC-3’ |

**Table S2.** Surface elements composition of PEEK after different surface modification

| Group | Composition | | | |
| --- | --- | --- | --- | --- |
|  | C% | O% | N% | N/C |
| PBS-PEEK | 80.77 | 18.53 | 0.71 | 0.00879 |
| Azide-DOPA_4_-PEEK | 68.2 | 28.44 | 3.36 | 0.04927 |
| BMP2p-PEEK | 70.07 | 24.64 | 5.29 | 0.07550 |
| DOPA_4_@BMP2p-PEEK | 61.98 | 30.69 | 7.33 | 0.11826 |


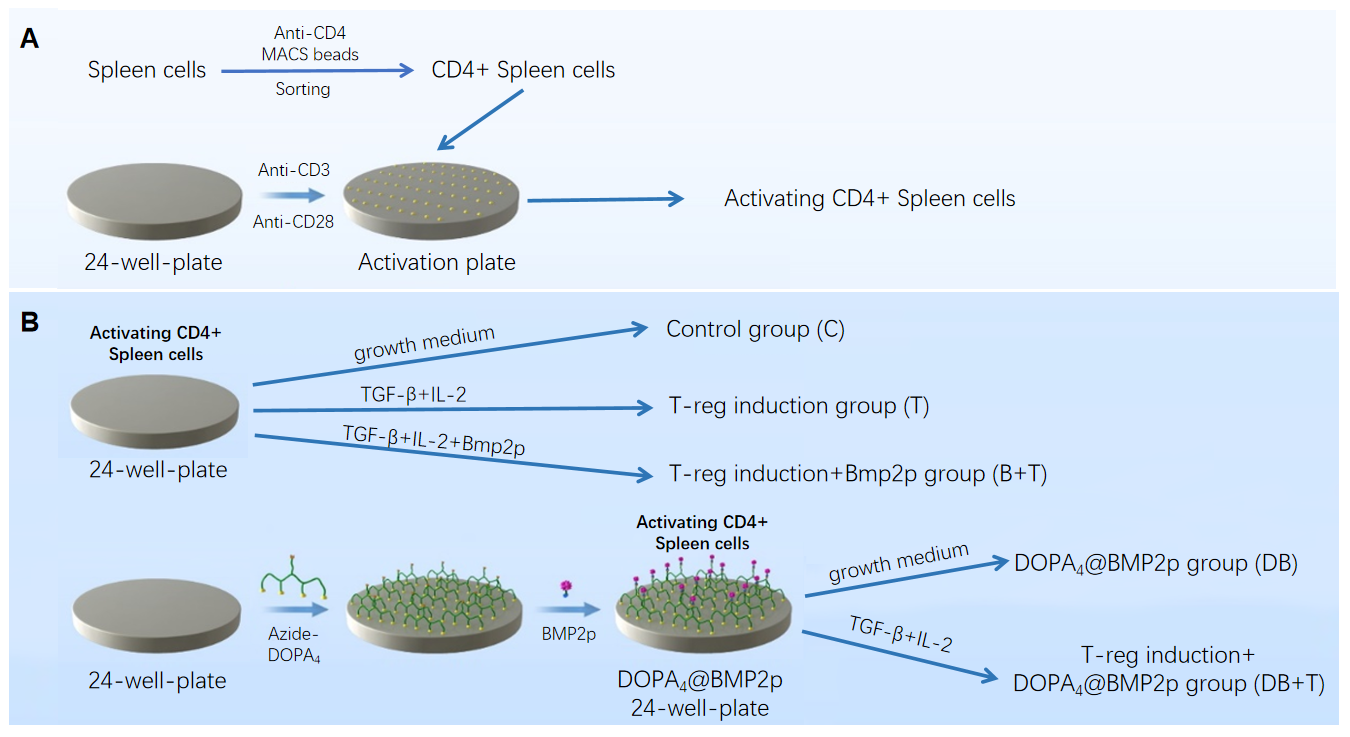


**Supplementary scheme 1.** Scheme of CD4+ spleen T cells isolation and Treg cells induction. (A) CD4+ spleen T cells isolation and activation, (B) Treg cells induction.


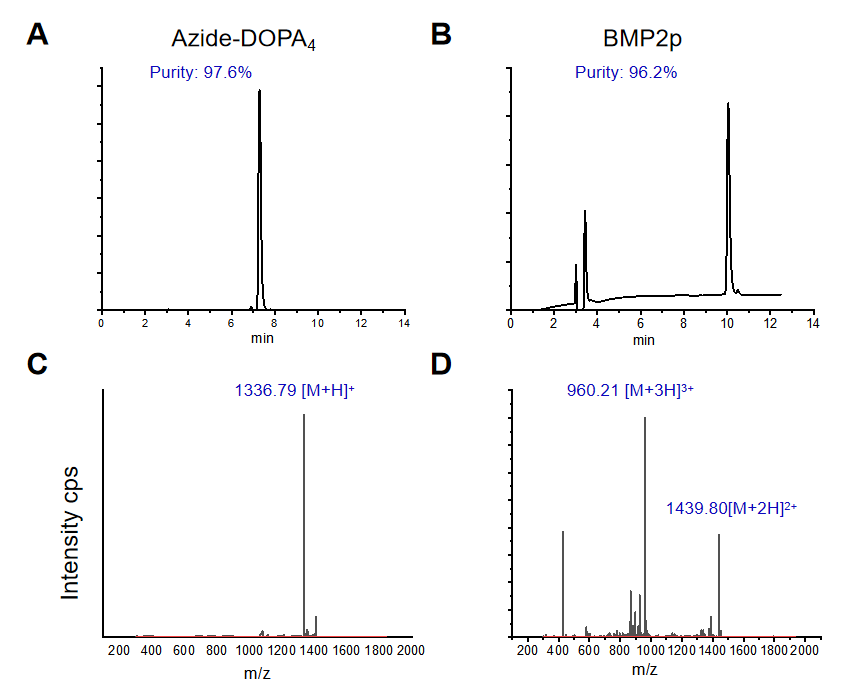


**Supplementary Figure 1.** Synthesis and identification of biomimetic peptides. (A-B) Purification of Azide-DOPA_4_ and DBCO-BMP2p by HPLC, (C-D) Identification of Azide-DOPA_4_ and DBCO-BMP2p by ESI-MS.


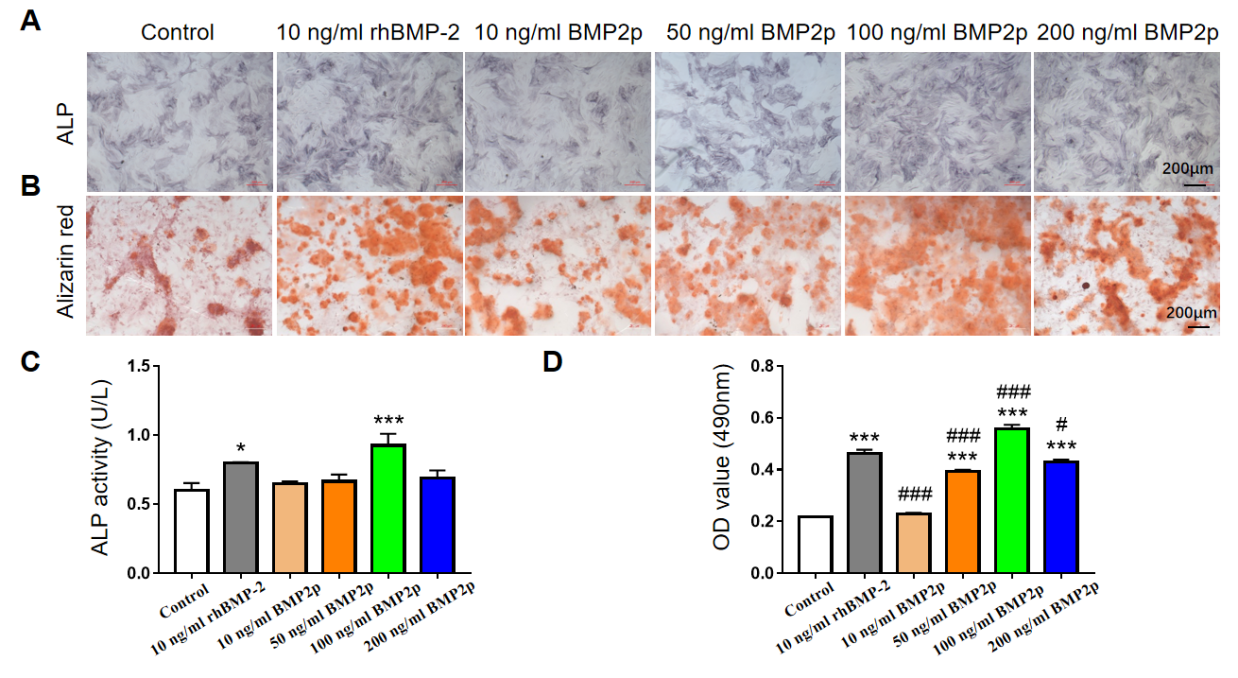


**Supplementary Figure 2.** In vitro osteogenesis induction capability of BMP2p. (A) Representative images of ALP staining of rBMSCs after 7 days of culture in osteogenic induction medium with different dose of BMP2p (10/50/100/200 ng/ml) or 10 ng/ml recombinant human bone morphogenetic protein-2 (rhBMP-2), cells cultured in osteogenic induction medium were set as control, (B) Representative images of Alizarin Red S staining after 14 days of cell culture, (C) and (D) Quantitative analysis of ALP activity and Alizarin Red S stained mineral layer. Statistically significant differences are indicated by *p < 0.05, ***p <0.001 compared with the control group, #p < 0.05, ###p <0.001 compared with the 10 ng/ml rhBMP-2 group.


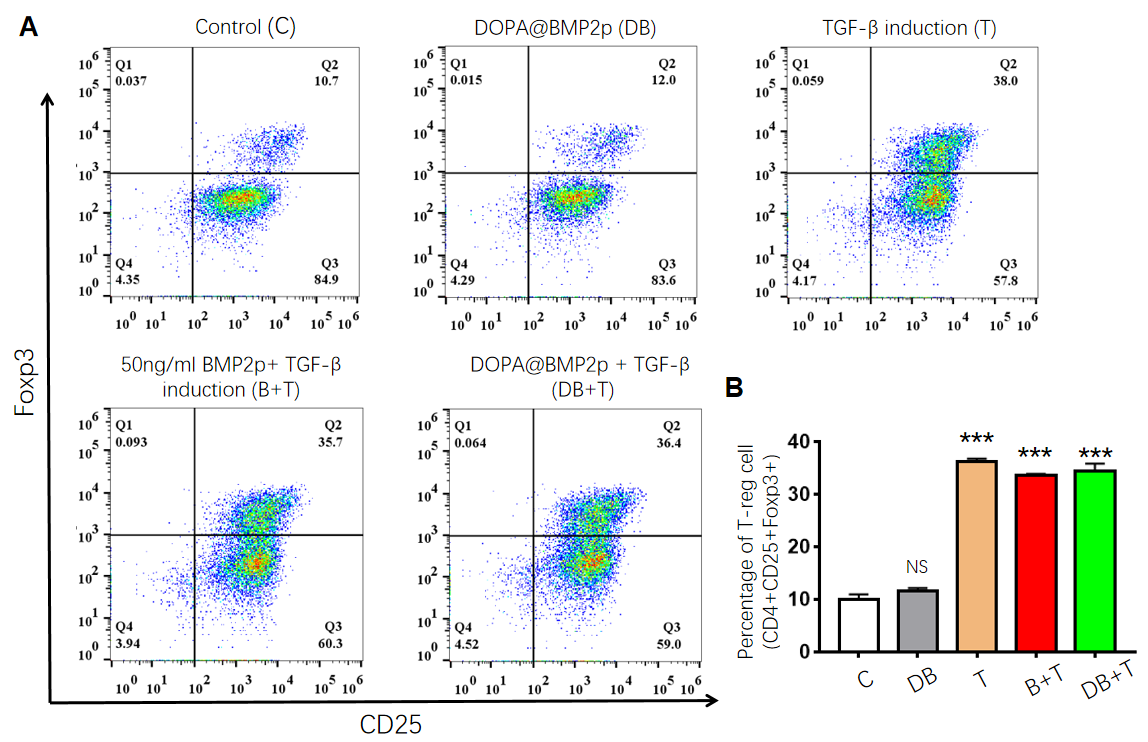


**Supplementary Figure 3.** Treg cells stimulation rate under different induction. (A) Flow cytometry analysis of activating CD4+ spleen cells under different induction, (B) Percentage of Treg cells (CD4+CD25+Foxp3+). Data are presented as the mean± SD, n=6. Statistically significant differences are indicated by ***p < 0.001 compared with control group (C) and NS means not statistically significant.
